# Falls Among Older Adults – Insights on Assessment and Prevention From Singapore and Thailand

**DOI:** 10.1093/geroni/igaf122.979

**Published:** 2025-12-31

**Authors:** Rahul Malhotra, Vanessa Koh

## Abstract

Falls, and fall-related injuries, are a major public health challenge for ageing populations worldwide; but especially in Asia, where the proportion of older adults, aged 60 years and older, has already surpassed the rest of the world. Yet, current international fall prevention guidelines are not tailored for Asian populations where significant ethnic, cultural and environmental differences in fall risk factors have been identified. This symposium informs fall assessment and prevention practice and policy for Asian populations by presenting five novel ongoing studies from two rapidly ageing Asian nations, Singapore and Thailand. The first presentation introduces TARGET, a Singapore-based prospective cohort of community-dwelling older adults designed specifically for assessing fall and fracture risk. Analyzing data from TARGET, the second presentation highlights the need for resilience-boosting interventions among older adults to improve functional and psycho-social outcomes. The third presentation introduces SAFE-TECH, a tailored, multi-component community-based fall prevention program developed in Singapore, aimed at improving physical performance, falls efficacy, and promoting preventive behaviors. From Thailand, the fourth presentation introduces the Thai-STEADI program, adapted from the CDC’s falls prevention model, utilizing community health workers and care managers to deliver scalable interventions in low-resource settings. The final presentation introduces a simulation model designed to identify policy bottlenecks and optimal strategies for implementation of a national falls prevention framework in Singapore. Together, this symposium discusses individual, community, and policy-level falls assessment and prevention strategies, and explores cross-country learnings for healthcare professionals, researchers, and policymakers. Aging Among Asians Interest Group Sponsored Symposium

